# ATTR-CM, HFpEF, and Clinical Cardiology: It Is Not a Moment, It Is a Movement

**DOI:** 10.1016/j.jaccao.2021.02.013

**Published:** 2021-08-17

**Authors:** Gautam S. Nayak

**Affiliations:** Cardiovascular Service Line, Confluence Health, Wenatchee, Washington, USA

**Keywords:** amyloidosis, heart failure, rural medicine

In 2015, a 66-year-old previously healthy man established care in my clinic in rural Washington. He was not feeling well, exhibiting signs of heart failure. An echocardiogram showed a normal ejection fraction, but a quite hypertrophied left ventricle, which did not seemingly correlate with his history or other objective data. Knowing the reticence a patient might feel about clinical ambiguity, I treaded lightly: “Let’s get a cardiac MRI to look at the heart muscle thickness in a bit more detail.” As his diagnosis came to light, I sent him to the University of Washington, where under the thoughtful guidance of an incredible team of heart failure experts, this patient’s evaluation culminated in a heart transplantation for transthyretin amyloid cardiomyopathy (ATTR-CM). To me, this was a seminal moment, and I suspect this story rings true for many cardiologists. Treating heart failure with preserved ejection fraction (HFpEF) had always been a bit frustrating knowing there was little we could do, but the power of just 1 patient success story seemed to open the floodgates for me. I was looking for amyloidosis everywhere, in every patient with even a modicum of signs. I was finding a fair share of cases and sending them on for advanced care, although the patients were much older and more frail and there was little we could do to really help them turn the tide of their disease. Eventually, an exasperated colleague in Seattle sighed, “What’s in the water out there?”

As I take in, with incredible awe and admiration, the wealth of data, diagnostics, and treatment for ATTR-CM, I try to frame it in the broader context of where we are in medicine. It really feels as though we’re on the cusp of an incredible movement in cardiology. More than 50% of patients with heart failure have HFpEF (1), yet there is little we can do to actually treat the condition. These patients have presented for years, and while most do not have amyloidosis, all are debilitated by a condition that reduces their lifespan, undermines their quality of life, and limits how they navigate their world. To be certain, some do improve with management of their chronic diseases, but treating a patient with HFpEF can often feel ungratifying, knowing the ultimate outcome was as bad as HFrEF, but with no guideline-directed medical therapy, cardiac resynchronization therapy–defibrillator, or MitraClip. In fact, many of my cardiology colleagues don’t treat HFpEF. “It’s really a primary care problem,” many lament as they prescribe Lasix and move on. Conditions like hypertension, obesity, diabetes, and a sedentary lifestyle drive much of the HFpEF milieu, but as we learn more about amyloidosis, it seems that our perspective on heart failure is transforming right before our eyes. The nexus of clinical recognition, highly specific noninvasive diagnostics, and transformative therapeutics have come together in a remarkable fashion.

Taking a step backward, and looking at where we are in the approach to cardiac amyloidosis has been so instructive. For example, my initial cases just a few years ago were typically diagnosed with a highly technical, advanced 2-hour diagnostic test (ie, cardiac magnetic resonance imaging), which then led to an endomyocardial biopsy at a transplant center. Treatment involved doxycycline and a clinical trial, if they qualified. Remarkably, the past few years have leveled the playing field for diagnostics in ATTR-CM, allowing community cardiology programs like mine to leverage local expertise in our cardiovascular team members to start more focused diagnostics for amyloidosis in our clinic. Software updates for transthoracic echocardiography now allow advanced strain rate imaging in any echocardiography lab, which can be a helpful screen for amyloidosis and help to differentiate it from other infiltrative or hypertrophic cardiomyopathies. Technetium pyrophosphate (PYP) nuclear scanning can be done in 20 minutes following the injection of a standard tracer used in bone scans. The specificity of PYP scans (2), has been empowering for our nuclear technicians, considering we spend hours every month reviewing our false-positive nuclear stress tests. Watching motivated echocardiographic and nuclear technicians embrace a new skill has been inspiring. They are learning novel techniques using the exact same tools they have expertly used for years, but toward a different end. We all recognized the impact ATTR had on patients too sick to travel to Seattle for tests, and keeping care local through our innovation has become a source of pride.

As a child of the 1980s who went to medical school in the late 1990s and trained in the early 2000s, I had the transformative experience of watching the acquired immunodeficiency syndrome (AIDS) epidemic evolve from a stigmatized death sentence to a chronic disease with remarkable therapeutics. I often reflect back on how those medical pioneers in San Francisco and New York must have felt when they made the connection between the rare Kaposi sarcoma and thrush they were seeing in gay men with what would eventually be traced to the human immunodeficiency virus (HIV). The ability to connect those dots in a minority population not only revolutionized infectious diseases and immunology, but it advanced the cause of health equity. Although early antiretrovirals for HIV were often disappointing, they moved the needle and laid a foundation for how we approach the development of diagnostics and therapeutics today well beyond HIV. Similarly, our approach to ATTR-CM has the potential to revolutionize how we approach more than 50% of patients with heart failure and perhaps even those who do not yet have it. We have seen this condition for years, but could not always connect the dots to make an actual diagnosis. My initial experience with ATTR-CM was not typical—it’s rare to see these patients get a transplant. Many are frail, elderly, and with comorbidities that often make more testing and treatment unappealing. As with HIV, the story of ATTR-CM is not about a small minority too sick to treat. Understanding this disease sets us on a path to preventing and eventually curing heart failure. Whether we call it diastolic heart failure, diastolic dysfunction, or HFpEF, the emerging recognition of amyloidosis will change how we view a previously vexing condition.

The ethical dilemmas of using the most expensive medications in the history of cardiovascular medicine to treat ATTR-CM is not lost on most of us, given the advanced age at which the diagnosis is often made. Transthyretin stabilizers are making a difference, and more therapeutics are in the pipeline. Although many patients can get these transformative medications at an affordable price or even free from the manufacturers, there are significant costs incurred to our stretched system. Engaging my pharmacy colleagues to dive into yet another expensive medication for a chronic condition as they grapple with authorizations for antiarrhythmics, novel diabetes agents, oral anticoagulants, antiplatelets, and vasodilators for many of our patients may feel like a bridge too far. Which battles are we going to fight? Ultimately, though, if we don’t keep advancing care for the sickest among us, we will lose the ability to treat or perhaps prevent HFpEF in the long term. It seems imperative to focus less on age and more on the disease itself, the prognosis, and what we can do to improve quality of life. Patients with HFpEF die of heart failure, and they grapple with life-limiting dyspnea, fatigue, and medication side-effects for decades. While most do not have amyloidosis, those that do are often relieved to know there is a reason. Just acknowledging there could be a cause to the symptoms, and doing basic tests to look for something previously unrecognized, gives hope. It allows us to overcome the clinical inertia we’ve had for a condition thought to be untreatable for decades. Notably, therapy for ATTR-CM may be most effective when administered before significant cardiac dysfunction (2), so early identification is important, and the more patients we identify, the better we get. If we don’t think about the diagnosis and look, we may be missing opportunities, even for those at a more advanced age. Each patient has their own unique value system and goals of care, like most with heart failure or other chronic conditions. I’ve been surprised at how open most of my elderly patients are to starting treatment for ATTR-CM, despite potential financial barriers, though I know the cost equation for these drugs has to be evaluated. We do our best, innovating in small ways for each case we find. It starts with ATTR-CM, but the opportunities to better understand heart failure become limitless when we evolve with our patients.

As a rural cardiologist caring for patients in a large geographic area, I don’t have the luxury of tailoring my practice to specific disease states. I travel throughout our region, see patients where they are, and find myself serving as both an internist and cardiologist. Our careers humble us daily, and seeing a bigger picture can be difficult when we’re navigating a full day of clinic or a busy inpatient service. What keeps me grounded is the recognition that smart people are thinking hard about the dilemmas I encounter daily, and they are using their expertise to create ways to help me care for all of my patients. The diagnosis and treatment of ATTR-CM did not just logically happen, although future guidelines will make it seem like a fait accompli. What we are seeing is the remarkable nexus of diagnostics and therapeutics coupled with a disease state that has always been there but never fully recognized. It feels like we should appreciate this moment, embrace what it means for our profession and our patients, and use it as a foundation to build a clinical care paradigm for a condition afflicting so many. It’s remarkable to me that as practicing clinicians in any location, we can leverage minimal local resources to diagnose and treat a form of HFpEF while learning new skills, elevating the careers of those around us, and, most importantly, helping our patients. Indeed, beyond the impact we have had on patients, the emergence of ATTR-CM in our local consciousness has allowed me an incredible opportunity to build a team of cardiovascular professionals equally dedicated to raising their game and making a difference.

I recently saw the ATTR-CM patient who received the heart transplant. Under the thoughtful care of his transplant team, his progress has been remarkable. I relayed to him that in the 5 short years since he and I met, the world of cardiovascular medicine has coalesced around ATTR-CM and advances in the disease state have been astounding. What felt most inspiring was that his success is now transforming the lives of countless others in our rural community, and that we can be a part of this clinical evolution in real time. As a mid-career physician, I’m old enough to know that this really is a moment, and experienced enough to realize that what I know now will probably seem quaint in a few years. I am grateful to my cardio-oncology and heart failure colleagues who are devoting their careers to moving the field forward and giving so many of our patients new hope. I am also lucky to work with a team in my rural cardiology practice committed to bringing clinical innovation to our underserved population—if we can do it in north central Washington, it can be done anywhere. The recognition, diagnostics, and treatment for cardiac amyloidosis is a parable for what comes next in managing heart failure, although most patients with HFpEF are still waiting for an effective treatment. We’re seeing a revolution in cardiovascular medicine right now, and it’s not confined to the advanced quaternary care centers. Everyone should feel empowered, no matter where you practice. The converging stories of ATTR-CM and HFpEF are just beginning, and from what I can tell, it’s going to be incredible.

## Funding Support and Author Disclosures

The author has reported that he has no relationships relevant to the contents of this paper to disclose.
